# Fibroblast growth factor 21 (FGF21) is robustly induced by ethanol and has a protective role in ethanol associated liver injury

**DOI:** 10.1016/j.molmet.2017.08.004

**Published:** 2017-08-19

**Authors:** Bhavna N. Desai, Garima Singhal, Mikiko Watanabe, Darko Stevanovic, Thomas Lundasen, ffolliott M. Fisher, Marie L. Mather, Hilde G. Vardeh, Nicholas Douris, Andrew C. Adams, Imad A. Nasser, Garret A. FitzGerald, Jeffrey S. Flier, Carsten Skarke, Eleftheria Maratos-Flier

**Affiliations:** 1 Department of Medicine, Beth Israel Deaconess Medical Center, Harvard Medical School, Boston, MA 02215, USA; 2 Department of Pathology, Beth Israel Deaconess Medical Center, Harvard Medical School, Boston, MA 02215, USA; 3 Eli Lilly and Company, Indianapolis, IN 46285, USA; 4 Department of Systems Pharmacology and Translational Therapeutics, Institute of Translational and Medicine and Therapeutics, University of Pennsylvania, Philadelphia, PA 19104, USA; 5 Department of Neurobiology, Harvard Medical School, Boston, MA 02215, USA

**Keywords:** Binge ethanol consumption, FGF21, Chronic ethanol consumption, Alcoholic liver disease, ACSS2, acyl-coenzyme a synthetase short-chain family member 2, ADH 1 and 2, alcohol dehydrogenase 1 and 2, ALDH1α1, aldehyde dehydrogenase 1 alpha 1, ALD, alcoholic liver disease, ALT, alanine aminotransferase, CD68, cluster of differentiation 68, ChREBPβ, carbohydrate-responsive element-binding protein beta, CPT1α, carnitine palmitoyltransferase 1 alpha, CPT1β, carnitine palmitoyltransferase 1 beta, CYP2E1, cytochrome P450 family 2 subfamily E member 1, FAS, fatty acid synthase, FGF21, fibroblast growth factor 21, FGF21-KO, mice lacking the FGF21 gene, FGF21-OE, mice overexpressing the FGF21 gene, IP, intraperitoneal, LDC, Lieber–DeCarli diet, MCP1, monocyte chemoattractant protein 1, NAFLD, non-alcoholic fatty liver disease, PPARα, peroxisome proliferator-activated receptor alpha, SC, subcutaneous, SCD1, stearoyl-CoA desaturase 1, SEM, standard error of the mean, SREBP1c, sterol regulatory element-binding protein-1c, TNFα, tumor necrosis factor alpha, UCP2, uncoupling protein 2, WT, wild type

## Abstract

**Objective:**

Excess ethanol consumption has serious pathologic consequences. In humans, repeated episodes of binge drinking can lead to liver damage and have adverse effects on other organs such as pancreas and brain. Long term chronic consumption of ethanol can also result in progressive alcoholic liver disease and cirrhosis. Fibroblast growth factor 21 (FGF21) is a metabolic regulator with multiple physiologic functions. FGF21 is a novel biomarker for non-alcoholic fatty liver disease (NAFLD) in humans and limits hepatotoxicity in mice. Therefore, we explored the possibility that FGF21 plays a role in response to ethanol consumption in both humans and mice.

**Methods:**

We used a binge drinking paradigm in humans to examine the effect of acute ethanol consumption on circulating FGF21. We adapted this paradigm to evaluate the acute response to ethanol in mice. We then examined the role of FGF21 on liver pathology in two models of chronic ethanol consumption in both wild type (WT) mice and mice lacking FGF21 (FGF21-KO).

**Results:**

Acute ethanol consumption resulted in a robust induction of serum FGF21 after 6 h in both humans and mice. Serum ethanol peaked at 1 h in both species and was cleared by 6 h. Ethanol clearance was the same in WT and FGF21-KO mice, indicating that FGF21 does not play a major role in ethanol metabolism in a binge paradigm. When FGF21-KO mice were fed the Lieber–DeCarli diet, a high fat diet supplemented with ethanol, a higher mortality was observed compared to WT mice after 16 days on the diet. When FGF21-KO mice consumed 30% ethanol in drinking water, along with a normal chow diet, there was no mortality observed even after 16 weeks, but the FGF21-KO mice had significant liver pathology compared to WT mice.

**Conclusions:**

Acute or binge ethanol consumption significantly increases circulating FGF21 levels in both humans and mice. However, FGF21 does not play a role in acute ethanol clearance. In contrast, chronic ethanol consumption in the absence of FGF21 is associated with significant liver pathology alone or in combination with excess mortality, depending on the type of diet consumed with ethanol. This suggests that FGF21 protects against long term ethanol induced hepatic damage and may attenuate progression of alcoholic liver disease. Further study is required to assess the therapeutic potential of FGF21 in the treatment of alcoholic liver disease.

## Highlights

• Binge ethanol consumption robustly increases circulating FGF21 levels in both humans and mice.
• FGF21 does not play a role in ethanol clearance which is the same in WT and FGF21-KO mice.
• In mice lacking FGF21, in two models of ethanol consumption there was either excess mortality or excess hepatic damage and inflammation.
• These data suggest the FGF21 may have potential therapeutic value for alcoholic liver disease.

## 1 Introduction

Over the last decade, fibroblast growth factor 21 (FGF21) has emerged as a critical metabolic regulator. The hormone is synthesized by multiple tissues including the liver, pancreas, adipose tissue, and muscle and exerts its action on several target tissues [1]. The FGF21 protein sequence is conserved across a wide range of mammalian species; however, its physiologic actions can be distinct. For example, hepatic expression of FGF21 rises dramatically in mice that are fasting or consuming a ketogenic diet and is accompanied by increased circulating FGF21 levels [2], [3]. In contrast, humans consuming ketogenic diets have no changes in serum FGF21 levels [4]. In addition, fasting in humans for 18–24 h also has no effect on serum levels, while fasting for 72 h in men is associated with a decline in circulating FGF21 [4]. Two studies examining longer term fasting in women for 7 and 10 days report increased FGF21 levels. As this is accompanied by higher levels of hepatic transaminases, this rise may represent a stress response [5].

In contrast to the discordant responses between mice and humans to fasting and ketogenic diets, hepatic fat accumulation leads to a consistent increase in FGF21 hepatic gene expression and a 3–4 fold increase in circulating levels, reported in both humans and mice [4], [6], [7]. Interestingly, the response of FGF21 to fructose is also similar in both mice and humans. In lean healthy humans and in individuals with metabolic syndrome, acute oral loading of fructose leads to a robust increase in circulating FGF21 within 2 h, which declines over the ensuing 5–6 h [8]. In mice, FGF21 is elevated after an acute gavage of fructose and also after fasting-refeeding with a high fructose diet; this increase appears to be mediated by the transcription factor ChREBPβ [9]. Similarly, protein restriction leads to increased FGF21 in both species [10]. In addition to responding to dietary manipulations and hepatic fat accumulation, FGF21 expression in the liver increases after exposure to hepatic stressors such as acetaminophen [11].

Chronic ethanol consumption is known to be lipotoxic and is associated with accumulation of hepatic fat [12]. While alcoholic liver disease (ALD) and non-alcoholic fatty liver disease (NAFLD) have distinct characteristics [13], they also share similar pathologies and can progress to fibrosis and cirrhosis and increase predisposition to hepatocellular carcinoma [14]. Considering the similar pathology observed in NAFLD and ALD, we hypothesized that ethanol consumption might also increase expression of FGF21.

We first tested this hypothesis in humans, measuring circulating FGF21 after binge ethanol consumption, which resulted in a 40-fold induction of serum FGF21 in humans 6 h after exposure to ethanol. A similar time course and robust increase in FGF21 levels was observed in mice orally gavaged with ethanol. Despite the increase observed, FGF21 did not appear to play a role in acute ethanol clearance or in ethanol induced changes in hepatic gene expression, as these were similar in WT and FGF21-KO mice.

In contrast, deletion of FGF21 was associated with significant hepatic pathology after long term exposure to ethanol. When ethanol was consumed in the context of a high fat diet, lack of FGF21 was associated with excess mortality within 16 days. After chronic exposure to ethanol in drinking water and a chow diet, WT mice demonstrated no obvious systemic adverse effects after 16 weeks. However, FGF21-KO mice showed increased hepatic injury. These data suggest that FGF21 plays a hepato-protective role in the context of chronic ethanol consumption.

As there are data suggesting that FGF21 plays a role in ethanol preference in both mice and humans [15], [16], we evaluated preference for ethanol in different FGF21 mouse models. In contrast to previous reports, we found no difference in ethanol preference between mice lacking FGF21 and WT mice, but did confirm that excess FGF21 leads to reduced consumption of ethanol.

## 2 Materials and methods

### 2.1 Human studies

To evaluate the effects of acute ethanol consumption on FGF21 in humans, biospecimens were sourced from the human study “Effects of Fish Oil and Red Wine on Oxidative Stress Biomarkers”, registered at clinicaltrials.gov as NCT00682318, aimed to evaluate the interaction between fish oil on oxidative stress markers after ethanol consumption [17], [18]. The clinical study was approved by the Institutional Review Board of the University of Pennsylvania (IRB# 807069) and by the Food and Drug Administration (FDA IND# 79,750; sponsor-investigator C.S.). Healthy non-smoking volunteers, n = 7 (4 females, 3 males), 38.4 ± 13.2 years, BMI < 25, with no history of alcohol dependence or liver disease were recruited through local advertisements. A summary of the inclusion criteria, exclusion criteria, and specific dietary restrictions before enrollment in the study is described in Supplementary Table 1. Subject characteristics are summarized in Supplementary Table 2. Routine medical history, physical exam, and laboratory work (hematology, biochemistry, and urinalysis) were assessed at screening and on completion of the study.

#### 2.1.1 Fish oil and alcohol study

In a longitudinal study design, volunteers received a placebo drink at baseline (diet *ad libitum*), followed by consumption of either a placebo drink or ethanol (0.4 g/kg body weight), randomized for order, after supplementation with 2 g/day of Lovaza, a synthetic prescription fish oil (omega-3-acid ethyl esters) for 5–6 weeks (41.9 ± 0.4 days). Subjects were then randomized to either continue Lovaza at a higher dose of 10 g/day, or were switched to receive Solgar, a safflower oil supplement at a dose of 10 g/day. After a mean 27.8 ± 0.5 days of supplementation subjects were challenged with ethanol at the higher dose of 0.9 g/kg body weight. As high doses of fish oil supplements increase exposure to heavy metals including mercury, participants were asked to refrain from additional fish foods during the study.

For each of the alcohol and placebo administrations an overnight fast was required, allowing water *ad libitum*. The morning after, volunteers were admitted to Center of Human Phenomic Science (CHPS) at the University of Pennsylvania approximately 2 h prior to ingestion of ethanol or placebo (approximately 07:00). The University of Pennsylvania Investigational Drug Service (IDS) prepared and dispensed all drinks using the body weight recorded during the screening visit. Body weight was again assessed prior to each alcohol/placebo administration to ensure that weight did not differ by more than 5% from initial weight. Alcohol drinks were prepared from 200-proof pharmaceutical grade purified ethanol. The ethanol content was calculated based on the appropriate dose for each subject adjusting for body weight, and sugar free lemonade (with/without additional sugar free fruit flavors) was used to dilute the drink to a total volume of 240 ml. Placebo and alcohol solutions were each provided in six Yorker-tip bottles, each bottle holding 40 ml. Subjects drank one bottle every 10 min, i.e. at 0, 10, 20, 30, 40, and 50 min, thus a total of 240 ml were ingested by each subject. Biospecimen collection was timed to occur at baseline and at 6 h and 24 h post ethanol/placebo administration. Compliance to ethanol/placebo administrations was assessed by direct oversight at the bedside as well as real-time monitoring blood ethanol levels with a breath analyzer.

Study participants received standardized meals at specified times: breakfast 2 h ± 1 h after the end of the ethanol/placebo administration, lunch and dinner 4 h ± 1 h and 6 h ± 1 h after the end of the ethanol/placebo administration, respectively.

#### 2.1.2 Binge ethanol challenge

The protocol described above was amended to obtain approval to measure serum FGF21 levels after administration of ethanol at 0.9 g/kg body weight in human subjects not receiving fish oil supplements and eating *ad libitum*. Blood was collected at baseline, then at 0.5, 1, 1.5, 2, 3, 4, 5, 6, 8, 10, and 24 h post administration. Volunteers were admitted to CHPS the night before the ethanol administration (16:30 ± 1:00 h) and fasted overnight with *ad libitum* access to water. Food intake during the inpatient stay was standardized to deliver low or no fructose, drinks without added sugar (e.g. milk, water, sugar free soda), and food low in carbohydrates (turkey and cheese sandwich on whole wheat bread supplemented with nuts, almonds, peanuts, and/or cheese and/or vegetables). The first meal subjects received on the day of ethanol administration was lunch at 13:00, while dinner was served at 18:00 on both days.

#### 2.1.3 Human serum analysis

Human FGF21 was measured using a commercially available Quantikine human FGF21 enzyme-linked immunosorbent assay (R&D Systems, Minneapolis MN). Glucose, triglycerides and alanine aminotransferase (ALT) activity were measured using the ACE Glucose Kit, ACE triglycerides kit and ACE ALT Kit respectively (Alfa Wasserman, West Caldwell NJ).

### 2.2 Animal studies

#### 2.2.1 Mouse maintenance and diets

All experiments were carried out on 8–16 week old male or female mice with the following genotypes: WT (C57BL/6J), FGF21-KO, and transgenic mice overexpressing FGF21 (FGF21-OE). For experiments only involving WT mice, mice were purchased from Jackson Laboratories (Bar Harbor ME). Originally, FGF21-KO and FGF21-OE mice were generated at Eli Lilly Research Laboratories (Indianapolis IN) and have been described previously [19], [20]. For experiments involving FGF21-KO or FGF21-OE mice, founder mice for each colony were backcrossed onto the C57BL/6J line at least 10 times before the initiation of these studies and were used with their littermate controls. FGF21+/− matings were used for all our experimental mice. Occasionally, one single generation of WT × WT and KO × KO breeders were used. Unless otherwise specified, mice were provided with *ad libitum* access to water and the standard animal facility chow diet (6.7% fat, 23.6% protein and 56% carbohydrate in the form of starch 29.4%, 2.5% sucrose) (LabDiet 5008 – Pharmaserv, Framingham MA). The mice were kept under a 12 h light: 12 h dark cycle and an ambient temperature of 22 ± 2 °C. All procedures were in accordance with National Institutes of Health Guidelines for the Care and Use of Animals and approved by the Institutional Animal Care and Use Committee at Beth Israel Deaconess Medical Center (Boston MA).

#### 2.2.2 Binge drinking protocol

Male WT mice were fasted for 2.5 h before receiving 3 gavages of either ethanol at 3.5 g/kg or an equivalent volume of distilled water. Mice were kept on a heating pad throughout the experiment to prevent hypothermia. Ethanol or water gavaged mice were sacrificed at each of the following time points after the first gavage – 1.5 h, 3 h, 6 h, 9 h. Serum and livers were collected and stored at −80 °C until analyzed. In a separate experiment, WT and FGF21-KO male mice were fasted for 2.5 h before receiving an intraperitoneal (IP) injection of 20% ethanol (v/v) and tail blood was collected for serum at the following time points – 0.5 h, 1 h, 2 h, 4 h.

#### 2.2.3 Models of chronic ethanol consumption

##### 2.2.3.1 Ethanol in liquid Lieber–DeCarli diet

Male WT and FGF21-KO mice were initially given *ad libitum* access to the liquid Lieber–DeCarli control diet (CTRL) without ethanol supplementation in order to acclimate to liquids. Thereafter, the mice received ethanol supplemented Lieber–DeCarli Diet (LDC) [21]. The CTRL diet had a caloric profile of 49% Carbohydrate, 15.1% Protein, 35.9% Fat with a total fiber content of 4.3% (Bio-Serv F1259SP, Flemington NJ). The LDC diet had a caloric profile of 35.5% Ethanol, 13.5% Carbohydrate, 15.1% Protein, 35.9% Fat with a total fiber content of 7.2% (Bio-Serv F1258SP, Flemington NJ). Liquid diets were prepared fresh every day. The ethanol concentration (w/v) was gradually increased from 2% (experimental Day 0) to 4% (Day 4) and finally 6% (Day 8). As ethanol content was increased in the diet, maltose dextrin content (Bio-Serv F3653, Flemington NJ) was adjusted to keep the diet isocaloric. Body weight and caloric intake were measured daily. The experiment was terminated on Day 16 as observed mortality reached 28% in the FGF21-KO-LDC group. Mice were sacrificed, and serum and livers were collected and stored at −80 °C until further analysis. A separate experiment was carried out only to determine the survival curve of WT and FGF21-KO mice on LDC with 4% ethanol (w/v).

##### 2.2.3.2 Consumption of ethanol in drinking water

Female WT and FGF21-KO mice were provided with *ad libitum* access to a chow diet and either water or 30% ethanol (v/v) for 16 weeks. Ethanol solution was freshly prepared every few days. Body weights were measured weekly and caloric intake was measured every day during week 1 and week 16. After 16 weeks, the mice were sacrificed, and serum and livers were collected and stored at −80 °C until further analysis.

#### 2.2.4 Two-bottle taste preference test

This experiment was performed on male WT mice, FGF21-KO mice, and FGF21-OE mice. Mice had access to two bottles (Kaytee Products, Chilton WI), containing either water or 10% ethanol (v/v) in water. The bottles were pre-autoclaved before the experiment and the position of the two bottles was interchanged each day to exclude position based learned bias. Water and ethanol intake were measured every day for 7 days. To assess the effect of exogenous FGF21, WT mice were infused FGF21 at a dose of 24 μg/day/animal (Eli Lilly, Indianapolis IN), using osmotic mini-pumps implanted subcutaneously (SC) as described previously [22]. The mice were then subjected to the same two bottle preference test as above for 3 days.

#### 2.2.5 Mouse serum and liver assays

Mouse FGF21 concentrations were measured using Quantikine Mouse FGF21 ELISA (R&D Systems, Minneapolis MN). Ethanol levels were measured using the EnzyChrom Ethanol Assay Kit (BioAssay Systems, Hayward CA), and triglycerides were measured using the Triglyceride Liquicolor Kit (StanBio Laboratory, Boerne TX). For liver triglyceride content, a Folch extraction [23] was first performed on 150 mg of frozen tissue before calorimetric analysis using the Triglyceride Liquicolor Kit (StanBio Laboratory, Boerne TX).

#### 2.2.6 Enzyme activity

Liver alcohol dehydrogenase activity was measured using the QuantiChromTM Alcohol Dehydrogenase Kit (BioAssay Systems, Hayward CA) as per the manufacturer's instruction. Serum ALT activity was measured using the ALT-SGPT Liquid Kit (Pointe Scientific, Canton MI).

#### 2.2.7 RNA extraction and quantitative real-time PCR

RNA was isolated from livers flash-frozen in liquid nitrogen using Direct-zol RNA MiniPrep kit (Zymo Research, Irvine CA). cDNA was made from isolated RNA using oligo (dt), random hexamer primers and reverse transcriptase QuantiTech RT Kit (Qiagen, Germantown MD). Quantitative PCR was performed using the 7800HT (Applied Biosystems, Foster City CA) thermal cycler and SYBR Green master mix (Applied Biosystems, Foster City CA). Relative mRNA abundance was calculated and normalized to levels of the housekeeping gene 36B4.

#### 2.2.8 Histological analysis

A portion of the left, right, and median hepatic lobes was removed and fixed in 10% formalin at 4 °C overnight. Paraffin embedding and sectioning was performed by the Histology Core at Beth Israel Deaconess Medical Center. Sections with a thickness of 5 μm were stained with Hematoxylin and Eosin for histological analysis, and Sirius Red to specifically assess for fibrosis. Slides were analyzed by two experienced liver pathologists (I.A.N. and H.G.V) in a double blind fashion. Each slide was graded based on the NAFLD Activity Score (NAS) [24], which looks at degree of steatosis, inflammation, and ballooning, as well as the METAVIR Score [25], which assesses the degree of fibrosis.

### 2.3 Data analysis

Data are shown as mean ± standard error of the mean (SEM). Time course experiments were analyzed for significant differences using a two-way ANOVA with repeated measures followed by Bonferroni's post-hoc test for individual comparisons. Single point measures for four-way studies were analyzed using a two-way ANOVA followed by Bonferroni's post-hoc analysis test for individual comparisons. Single point measures for two-way studies were analyzed using a two-tailed unpaired t-test. *Significance is designated by asterisks with* *P < *0.05*, **P < *0.01*, ***P < *0.001*, ****P < *0.0001*, *or designated by letters where means that do not share a common letter are significantly different from each other at P* < *0.01.*

## 3 Results

### 3.1 Binge ethanol consumption induces FGF21 in humans in a time and dose dependent manner

**Figure 1:**
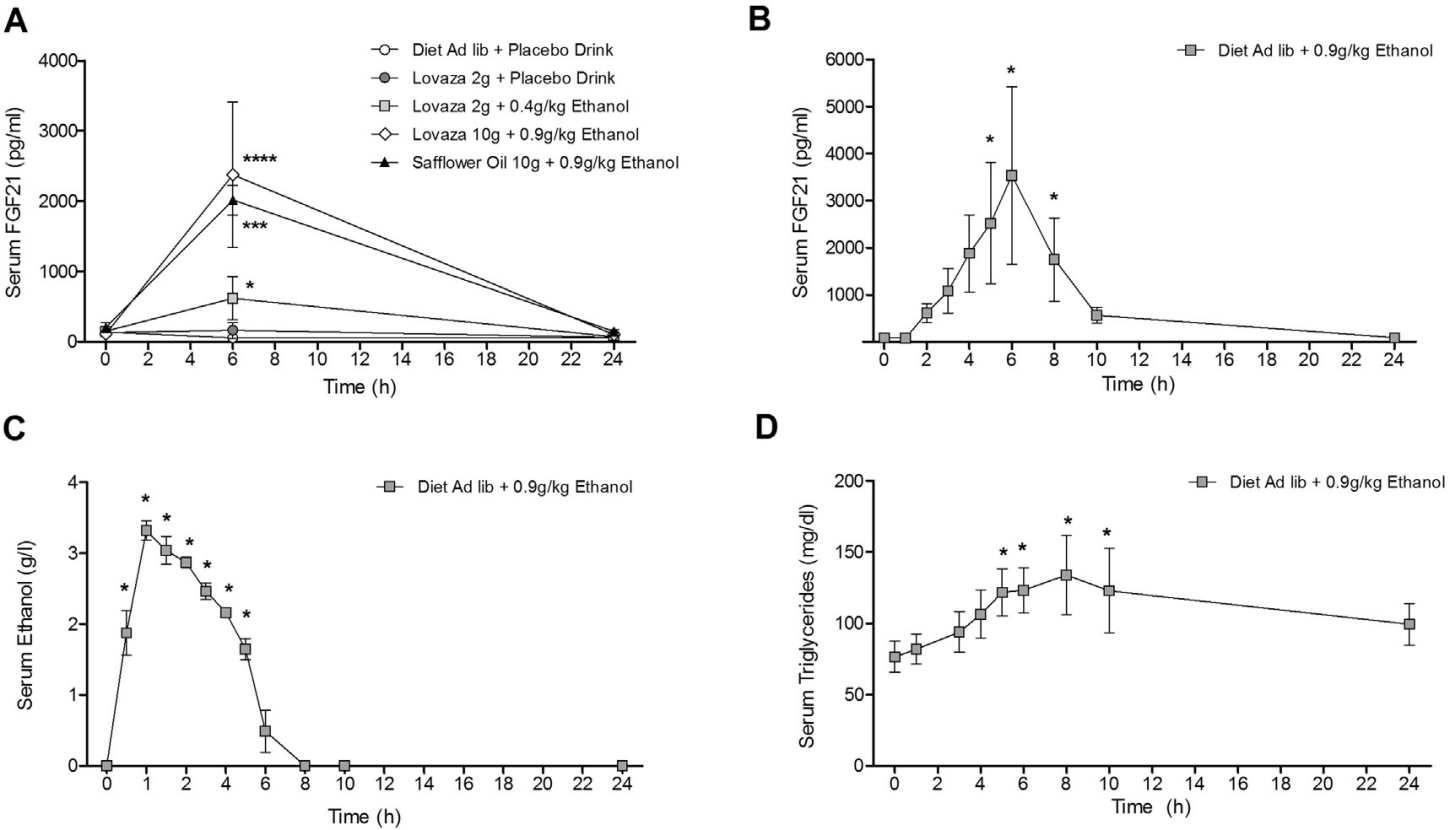
**Binge ethanol consumption in humans increases circulating FGF21 levels**. Binge consumption of ethanol elevates FGF21 in humans. In subjects receiving fatty acid supplements, a placebo drink has no effect on circulating FGF21 (A). Binge consumption of the lower ethanol dose (0.4 g/kg) in subjects receiving supplements causes an 11-fold increase in circulating FGF21 at 6 h. Remarkably, binge consumption of a higher ethanol dose (0.9 g/kg) results in a dramatic 40-fold elevation in circulating FGF21 at 6 h in these subjects (A). This effect is also consistent in subjects not receiving any fatty acid supplements, where the higher ethanol dose (0.9 g/kg) causes a similar 40-fold rise in circulating FGF21 at 6 h (B). Serum FGF21 levels increase over the first 6 h and then decline, returning to normal at 24 h (B). In these same subjects, serum ethanol peaks 1 h after the binge episode and is cleared from the circulation by 8 h (C). Serum triglycerides are elevated between 5 and 10 h after the ethanol challenge (D). *Data represented as Mean* ± *SEM*; *n* = *4*–*11 subjects*. *Significance was determined with a two-way ANOVA with repeated measures followed by a post-hoc analysis using Bonferroni's test*. *Significance is designated by asterisks with* *P < *0.05*, **P < *0.01*, ***P < *0.001*, ****P *<* *0.0001.*

**Supplementary Figure 1:**
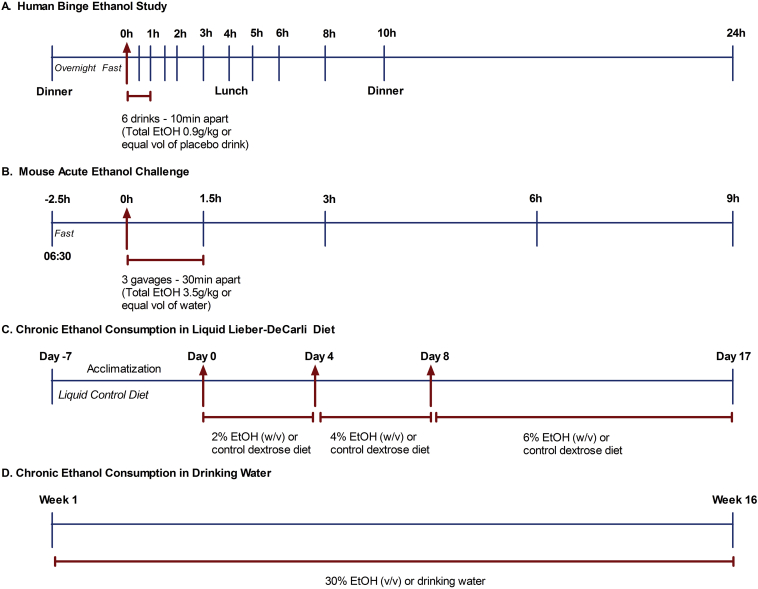
**Experimental design for acute and chronic ethanol consumption studies**. Study design for binge ethanol consumption in human subjects (A) and an adapted experimental paradigm of acute ethanol feeding in mice (B). Experimental design for mice chronically consuming ethanol supplemented in a high fat liquid LDC diet (C). Experimental design for mice chronically consuming ethanol in drinking water combined with a chow diet (D).

**Supplementary Figure 2:**
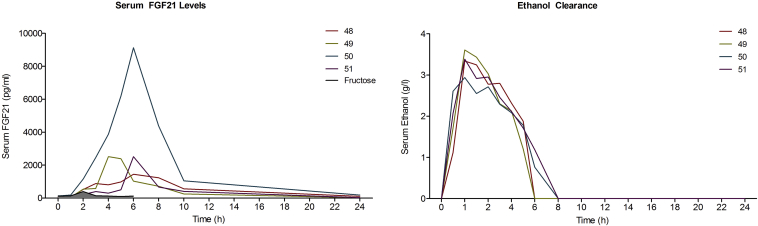
**Serum FGF21 and ethanol response in individual human subjects after binge consumption of ethanol**. Binge consumption of ethanol in healthy human subjects (0.9 g/kg) results in a significant elevation of serum FGF21 in each subject compared to the 3–4 fold induction observed with a 75 g fructose challenge (adapted from reference 8) (A). The time course of serum ethanol clearance in the individual subjects is the same (B).

Data from the first cohort, which was enrolled in the crossover study, showed that fatty acid supplements alone did not elevate FGF21 levels, regardless of the supplement type and dose (Figure 1A). Baseline serum FGF21 was 55 ± 14 pg/ml and is consistent with previous reports [4], [6]. Following binge ethanol consumption, FGF21 levels were dramatically elevated in comparison to subjects consuming a placebo drink (Figure 1A). The peak in FGF21 was proportional to the amount of ethanol consumed (Figure 1A) as subjects receiving 0.4 g/kg ethanol peaked at 700 ± 348 pg/ml (P = 0.03), while subjects receiving 0.9 g/kg ethanol peaked at 2194 ± 328 pg/ml (P < 0.0001). To further evaluate the temporal relationship between ethanol consumption and FGF21 serum levels in humans, we added a cohort of 4 subjects who did not receive fatty acid supplements (Experimental design: Supplementary Figure 1A). These subjects demonstrated a 40-fold increase in serum FGF21 levels 6 h after the ethanol challenge (FGF21 levels – 0 h: 88 ± 22 pg/ml; 6 h: 3536 ± 1890 pg/ml) (P = 0.05) (Figure 1B). The fold increase in FGF21 and the precise timing of the peak levels between subjects was somewhat variable (Supplementary Figure 2A), reiterating earlier observations on the inter-individual differences of FGF21 induction after a fructose challenge [8]. We then examined ethanol clearance. Serum ethanol peaked 1 h after the ethanol challenge in all subjects and was cleared from the system within 8 h (P < 0.05) (Figure 1C, Supplementary Figure 2B). Over the ensuing 24 h, serum triglycerides increased at 5 h and remained elevated until 10 h after the ethanol challenge (P < 0.05) after which they returned to baseline (Figure 1D). Glucose stayed within the physiological range, between 80 and 120 mg/dl (0 h: 92 ± 3 mg/dl; 6 h: 112 ± 11 mg/dl; 24 h: 94 ± 3 mg/dl). ALT levels in the serum also remained unchanged within the physiological range (0 h: 8 ± 0.6 IU/l; 6 h: 7.5 ± 0.9 IU/l; 24 h: 7.7 ± 1.5 IU/l).

### 3.2 Acute ethanol consumption induces FGF21 in wild type mice in a time dependent manner

**Figure 2:**
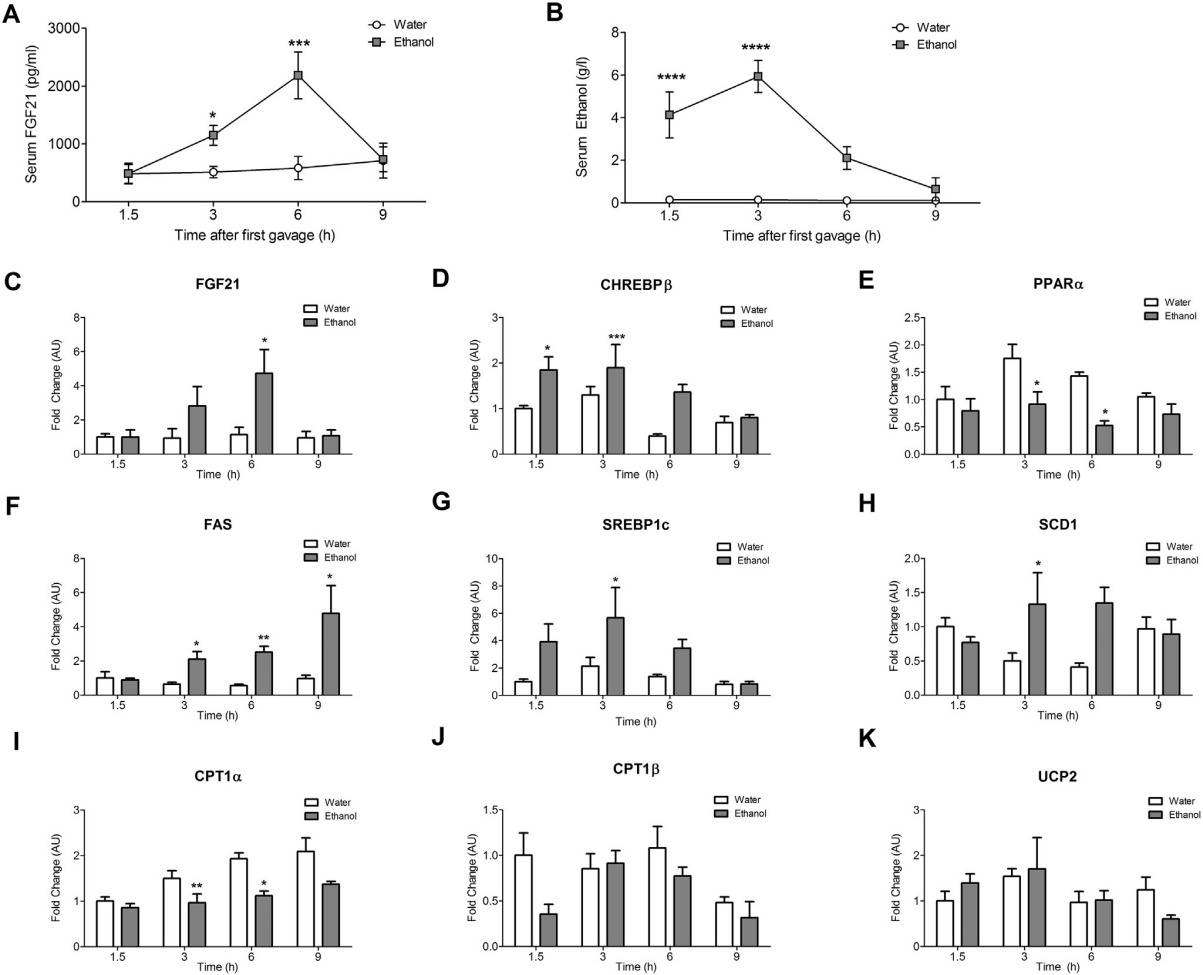
**Acute ethanol feeding in mice increases circulating FGF21 levels**. WT mice gavaged with ethanol have increased serum FGF21 levels that peak at 6 h (A). Serum ethanol levels peak at 1.5 h and are sustained until 3 h (B). Ethanol gavage in WT mice causes a time dependent increase in hepatic FGF21 gene expression (C). Carbohydrate induced transcription factor ChREBPβ is upregulated (D), and a simultaneous downregulation of transcription factor PPARα and its target is observed (E, K). Fatty acid synthesis genes are upregulated (F, G, H) whereas fatty acid oxidation genes are downregulated or remain unchanged (I, J). *Data represented as Mean* ± *SEM; n* = *8*–*10 mice*/*group*. *Significance in* (*A*) *and* (*B*) *was determined with a two-way ANOVA with repeated measures followed by a post-hoc analysis using Bonferroni's test*. *Significance in (C)–(K) was determined with a two-way ANOVA with Bonferroni's post-hoc test for individual comparisons. Significance is designated by asterisks with* *P < *0.05*, **P < *0.01,* ***P < *0.001*, ****P < *0.0001.*

As observed in humans, acute ethanol exposure in male WT mice correlated with a significant time dependent increase in circulating FGF21 (Experimental Design: Supplementary Figure 1B). FGF21 significantly increased 3 h after the first gavage, peaked at 6 h, and returned to baseline by 9 h (P = 0.05, P = 0.003) (Figure .2A). Serum ethanol levels in WT mice gavaged with ethanol peaked at 1.5 h, remained elevated at 3 h, and were cleared from the circulation or metabolized within 9 h after the first gavage (P < 0.0001) (Figure 2B).

Hepatic expression of FGF21 mRNA was increased at 6 h after the first gavage (P < 0.03) (Figure 2C). Transcription factor ChREBPβ (Figure 2D) was induced at 1.5 h and 3 h following ethanol gavage (P < 0.001). In contrast, expression of the transcription factor peroxisomal proliferator-activated receptor alpha (PPARα) was markedly reduced at both 3 h and 6 h after the first ethanol gavage (P < 0.05) (Figure 2E).

An increased expression of fatty acid synthesis genes was observed 6 h after acute ethanol gavage in WT mice, namely fatty acid synthase (FAS) (P < 0.01), sterol regulatory element-binding protein-1c (SREBP-1c) (P < 0.05) and steroyl-CoA desaturase 1 (SCD1) (P < 0.05) (Figure 2F–H). Simultaneously, there was a time dependent reduction in expression of fatty acid oxidation gene carnitine palmitoyl transferase 1 alpha (CPT1α) (P < 0.01), (Figure 2I), while fatty acid oxidation gene carnitine palmitoyl transferase 1β (CPT1β) and PPARα target gene uncoupling protein 2 (UCP2) remained unchanged (Figure 2J and K).

### 3.3 Chronic ethanol consumption in Lieber–DeCarli diet leads to excess mortality and hepatotoxicity in FGF21-KO mice

**Figure 3:**
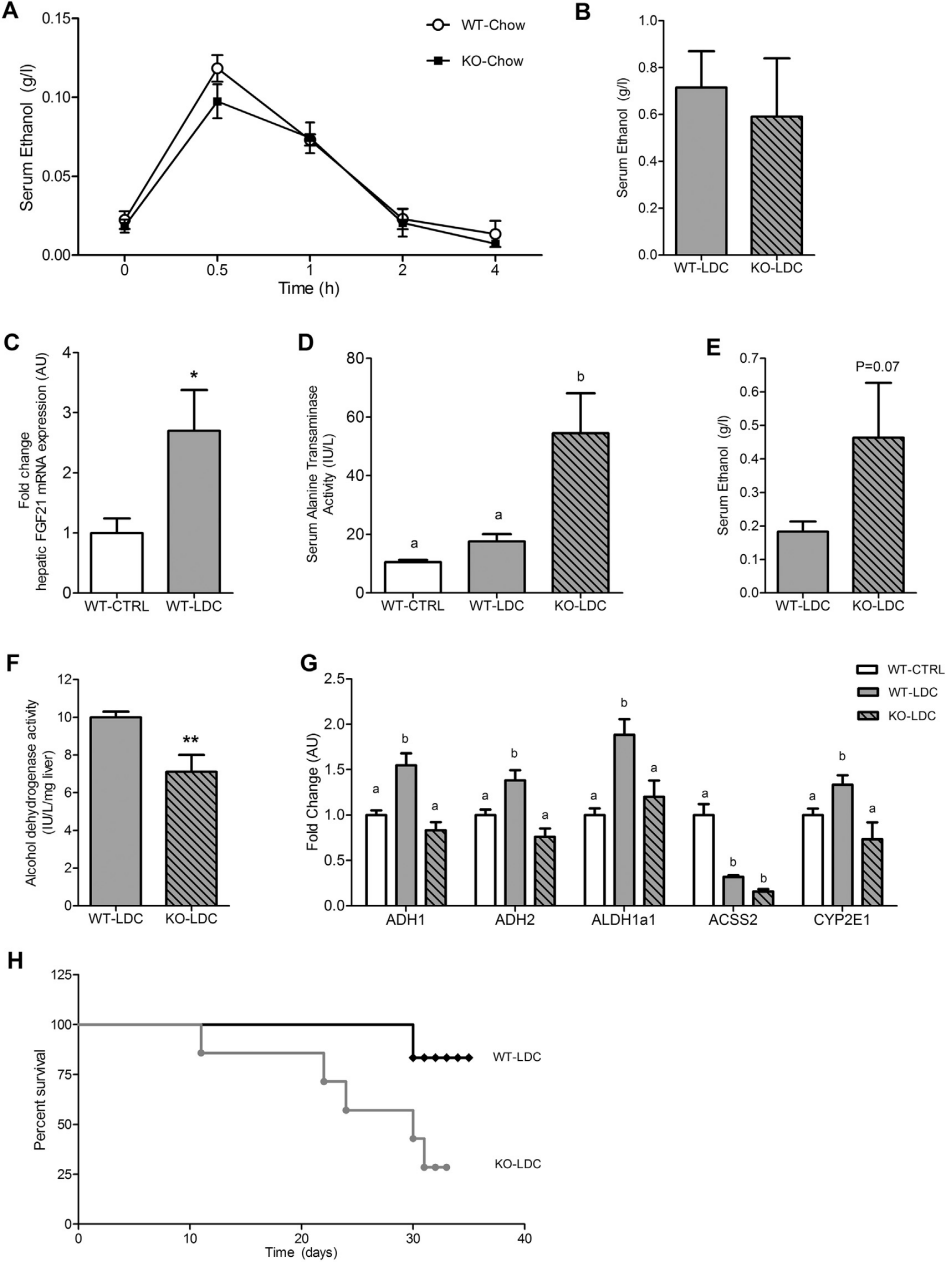
**Chronic ethanol consumption along with a Lieber**–**DeCarli diet leads to excess mortality and disruption of ethanol metabolism in FGF21-KO mice**. Clearance of ethanol after a single IP injection (20% v/v) is identical in WT and FGF21-KO mice consuming chow (A). Following a 12 h fast, WT and FGF21-KO mice fed ethanol in the Lieber–DeCarli (LDC) diet (4% w/v) have similar serum ethanol levels 4 h post re-feeding (B). Chronic ethanol consumption in LDC diet (6% w/v) for 16 days results in significant elevation of hepatic FGF21 gene expression in WT mice (C). Serum marker of hepatic damage ALT is elevated only in FGF21-KO mice on the LDC diet (D). Serum ethanol levels are elevated in FGF21-KO mice on the LDC diet for 16 days (E). Hepatic capacity for oxidizing ethanol is lower in FGF21-KO mice on the LDC diet, as measured by alcohol dehydrogenase activity (F). Expression of ethanol metabolizing enzymes is attenuated in FGF21-KO mice compared to WT mice on the LDC diet (G). A survival curve experiment in mice consuming ethanol in the LDC diet (4% w/v) reveals excess mortality in FGF21-KO mice on the LDC diet (H). *Data represented as Mean* ± *SEM; n* = *6*–*8 mice/group. Significance in* (*A*) *determined with a two-way ANOVA with repeated measures followed by a post-hoc analysis using Bonferroni's test. Significance in* (*B*), (*C*), (*E*), (*F*) *was determined with a unpaired two tailed student t-test. Significance in* (*H*) *was determined with a log rank* (*Mantel–cox*) *test. Significance in* (*D*), (*G*) *was determined with a two-way ANOVA and Bonferroni's post-hoc analysis test for individual comparisons. Significance is either designated by asterisks with* *P < *0.05,* **P < *0.01, or is designated by letters where means that do not share a common letter are significantly different from each other at P* < *0.01.*

Before starting the chronic experiments, baseline ethanol clearance rates were measured in WT and FGF21-KO male mice consuming a chow diet. After a single IP injection of ethanol (20% v/v), peak levels and ethanol clearance/metabolism rates were the same in WT and FGF21-KO mice. Serum ethanol levels peaked 0.5 h post injection and were cleared within 2 h (Figure 3A). In another experiment, mice were fasted for 12 h followed by refeeding with the liquid Lieber–DeCarli (LDC) diet (4% ethanol w/v); and serum ethanol and serum ALT levels were measured 4 h after the diet was made available. Both serum ethanol (Figure 3B) and ALT levels (WT-LDC: 18.0 ± 1.5 IU/l; KO-LDC: 22.1 ± 1.9 IU/l) were the same in both groups indicating that the acute response to ethanol is not affected by FGF21.

We first evaluated the role of FGF21 in chronic ethanol consumption using the LDC diet. WT and FGF21-KO mice were gradually exposed to the LDC diet, progressively increasing ethanol content to 2, 4, and 6% (w/v) (Experimental Design: Supplementary Figure 1C). At 6% ethanol concentration, we noted a mortality of 28% in FGF21-KO mice by day 16. Therefore, the remaining animals were sacrificed and livers were subsequently analyzed. Chronic ethanol consumption in the LDC diet led to a significant increase in hepatic FGF21 expression in WT-LDC mice compared to the WT-CTRL group (WT-CTRL: 1.0 AU ± 0.2 vs WT-LDC: 2.7 ± 0.7 AU; P = 0.03) (Figure 3C). Hepatocyte damage was observed specifically in FGF21-KO-LDC mice, as indicated by elevated serum ALT (WT-LDC: 17.60 ± 2.4 IU/l; FGF21-KO-LDC: 54.51 ± 13.6 IU/l, P = 0.01) (Figure 3D). Significantly higher serum ethanol levels were noted in FGF21-KO-LDC mice compared to WT-LDC mice after 16 days (Figure 3E). This was consistent with the lower activity of alcohol dehydrogenase which metabolizes ethanol to acetaldehyde (WT-LDC: 9.9 ± 0.3 IU/l/mg liver; FGF21-KO-LDC: 7.1 ± 0.9 IU/l/mg liver; P = 0.008) (Figure 3F). Ethanol metabolizing pathways are limited to a few oxidizing enzymes. Hepatic gene expression of alcohol dehydrogenase 1 and 2 (ADH1, 2), acetaldehyde dehydrogenase1a1 (ALDH1a1), acyl-coenzyme A synthetase short-chain family member 2 (ACSS2), which catalyzes acetaldehyde conversion to acetate, and cytochrome P450 family 2 subfamily E member 1 (Cyp2E1), a mitochondrial enzyme that metabolizes ethanol (Figure 3G), were all increased in WT mice on the LDC diet compared to WT mice on the CTRL diet. Induction of these genes was significantly attenuated in FGF21-KO mice on the LDC diet (Figure 3G) and was associated with increased accumulation of ethanol in the serum (Figure 3E).

To better assess the toxic effect of ethanol in FGF21-KO mice, survival rate was determined in these mice while consuming 4% LDC. Excess unexplained mortality was observed in FGF21-KO-LDC mice compared to WT-LDC mice as observed over 33 days (Percent survival of WT-LDC: 83.3%; Percent survival of FGF21-KO-LDC: 28.5%, P = 0.04) (Figure 3H). These results demonstrate that chronic ethanol exposure in the context of a high fat LDC diet leads to excess mortality in FGF21-KO mice compared to WT mice. Deficiency of FGF21 is associated with limited induction of ethanol oxidizing genes possibly contributing to the increased mortality.

**Figure 4:**
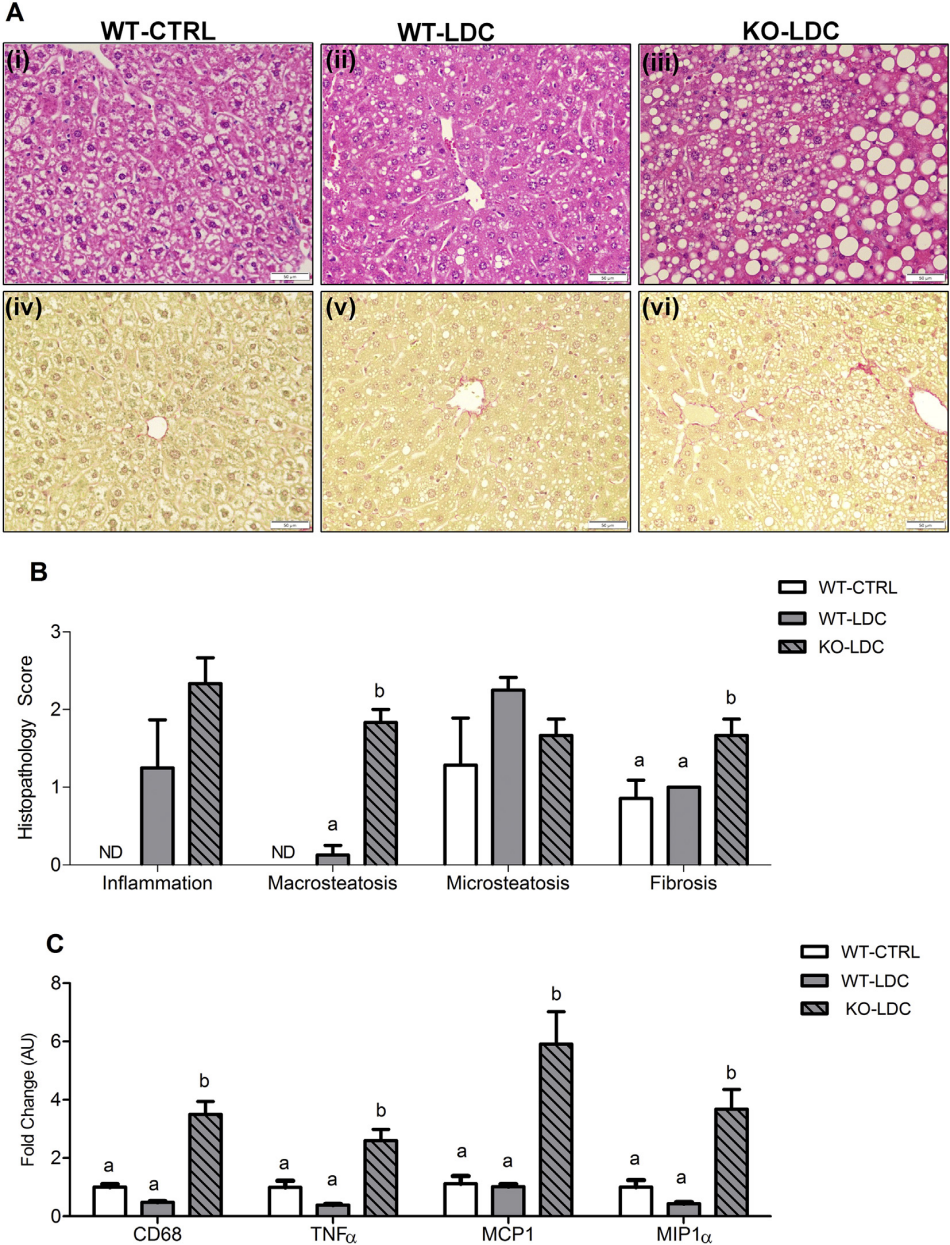
**FGF21-KO mice develop worse liver pathology when consuming ethanol in the Lieber–DeCarli diet**. Hematoxylin & Eosin staining of liver sections demonstrate relatively normal histology in WT mice consuming the CTRL diet or the LDC diet (A i–ii). In contrast, severe steatosis is observed in FGF21-KO on the LDC diet (A iii). Sirius Red staining reveals pronounced fibrosis in FGF21-KO-LDC mice (A vi) compared to WT-CTRL or WT-LDC mice (A iv–v). Double blind histopathological scoring confirms the excess liver damage in FGF21-KO-LDC mice, as indicated by specific macrosteatosis and fibrosis scores (B). In addition, FGF21-KO-LDC mice also have higher expression of inflammatory markers compared to WT-CTRL or WT-LDC mice (C). *Data represented as Mean* ± *SEM; n* = *6*–*8 mice*/*group. Significance in* (*B*) *and* (*C*) *was determined with a two-way ANOVA and Bonferroni's post-hoc analysis test for individual comparisons. Significance is designated by letters where means that do not share a common letter are significantly different from each other at P* < *0.01. Scale: 50 μm* (*A*).

Histological examination of livers showed relatively normal liver in WT-CTRL and WT-LDC mice (Figure 4A i, ii, iv, v). However, very prominent and severe steatosis and fibrosis were observed in FGF21-KO-LDC mice (Figure 4A iii, vi). Histopathological scoring confirmed severe macrovesicular steatosis and fibrosis in FGF21-KO-LDC mice (Figure 4B). Ballooning degeneration and hypertrophic changes were not observed in histology. Pathological analysis did not reveal any obvious signs of sudden mortality such as hemorrhage or congestion.

Hepatic gene expression of pro-inflammatory cytokines, cluster of differentiation 68 (CD68), tumor necrosis factor α (TNFα), chemokine monocyte chemoattractant protein 1 (MCP1), and macrophage Inflammatory Proteins-1α (MIP1α) was significantly elevated in FGF21-KO mice exposed to LDC compared with WT mice on the diet (Figure 4C).

### 3.4 Chronic ethanol consumption in drinking water leads to moderate hepatic damage in FGF21-KO mice

**Supplementary Figure 3:**
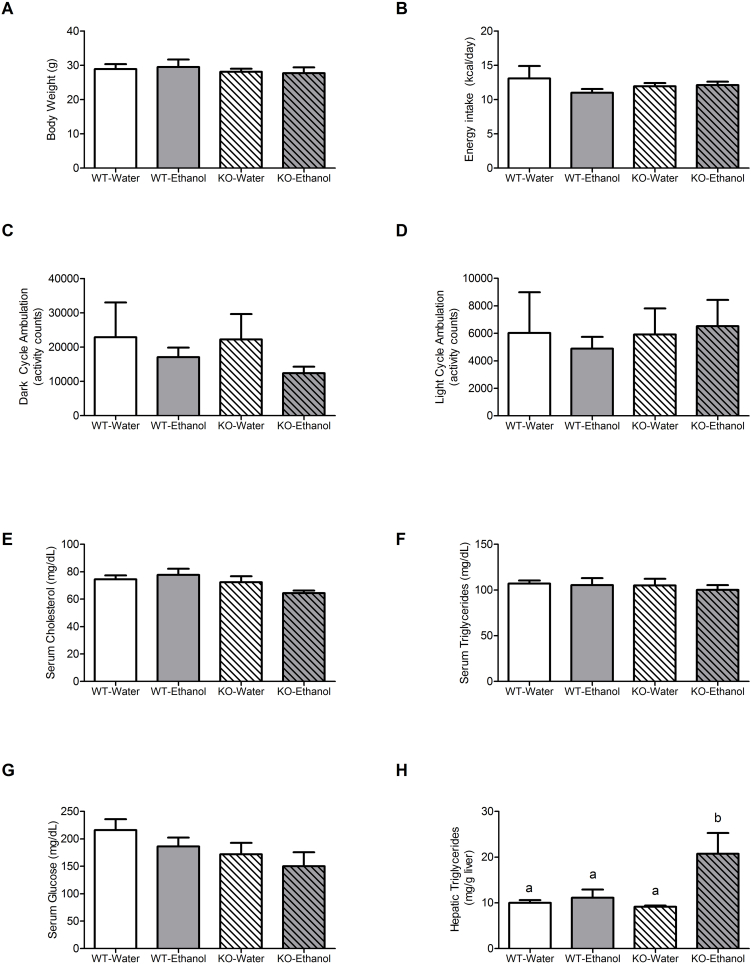
**Baseline parameters in mice chronically consuming ethanol in drinking water**. After 16 weeks, WT and FGF21-KO mice consuming 30% ethanol in drinking water along with a chow diet have similar body weights (A), caloric intake (B), locomotor activity (C, D), serum cholesterol (E), serum triglycerides (F), and serum glucose (G). In contrast, FGF21-KO-Ethanol mice have increased accumulation of triglycerides in the liver compared to other groups (H). *Data represented as Mean* ± *SEM; n* = *5*–*6 mice*/*group. Significance was determined with a two-way ANOVA and Bonferroni's post-hoc analysis test for individual comparisons. Significance is designated by letters where means that do not share a common letter are significantly different from each other at P* *<* *0.01.*

Since the LDC diet model was associated with significant mortality in FGF21-KO, we selected an alternative model to study the potential effects of FGF21 in chronic ethanol consumption. Female mice were exposed to 30% ethanol (v/v) in drinking water and a normal chow diet for 16 weeks (Experimental Design: Supplementary Figure 1D). Terminal body weight, caloric intake, and locomotor activity were not different within and between the groups (Supplementary Figure 3A–D), and no excess mortality was observed.

**Figure 5:**
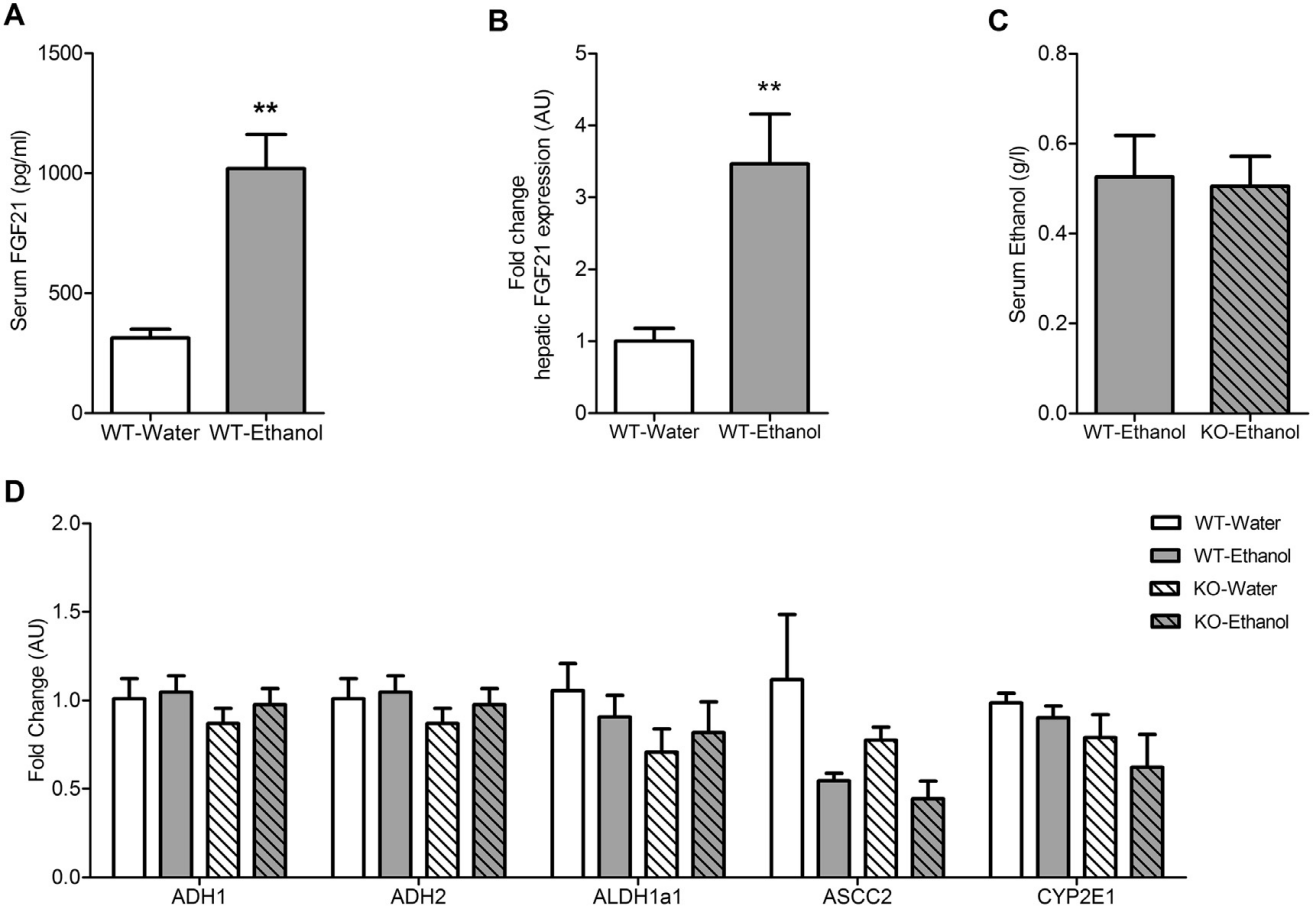
**Chronic ethanol consumption in drinking water along with a chow diet has no adverse effect on the ethanol metabolizing capacity of FGF21 KO mice**. WT mice consuming 30% ethanol in drinking water (v/v) and a chow diet for 16 weeks have significantly elevated serum FGF21 levels (A) and increased hepatic FGF21 expression (B). After 16 weeks, serum ethanol levels are similar in WT and FGF21-KO mice consuming ethanol (C). Hepatic gene expression of ethanol oxidizing enzymes is the same in all groups (D). *Data represented as Mean* ± *SEM; n* = *5*–*6 mice*/*group*. *Significance in* (*A*), (*B*), (*C*) *was determined with a two-tailed unpaired student t-test*. *Significance in* (*D*) *was determined with a two-way ANOVA and Bonferroni's post-hoc analysis test for individual comparisons*. Significance is designated by asterisks with **P < *0.01.*

Serum FGF21 was higher in WT mice consuming ethanol (WT-water: 312.8 ± 37.4 pg/ml; WT-Ethanol: 1020 ± 142.1 pg/ml; P = 0.003) as was hepatic FGF21 expression (WT-water: 1.0 ± 0.17 AU; WT-Ethanol: 3.46 ± 0.69 AU; P = 0.006) (Figure 5A and B). Since ethanol is cleared rapidly, serum was sampled 3 h into the dark cycle (21:00 h), when the mice are expected to be actively feeding and drinking. Female WT and FGF21-KO mice had identical serum ethanol levels indicating that FGF21-KO mice have no excess ethanol accumulation in the blood (Figure 5C). Hepatic gene expression of ethanol metabolizing enzymes were found to be very similar across groups (Figure 5D). Serum cholesterol, serum triglycerides, and serum glucose levels remained unchanged (Supplementary Figures 3E–G).

**Figure 6:**
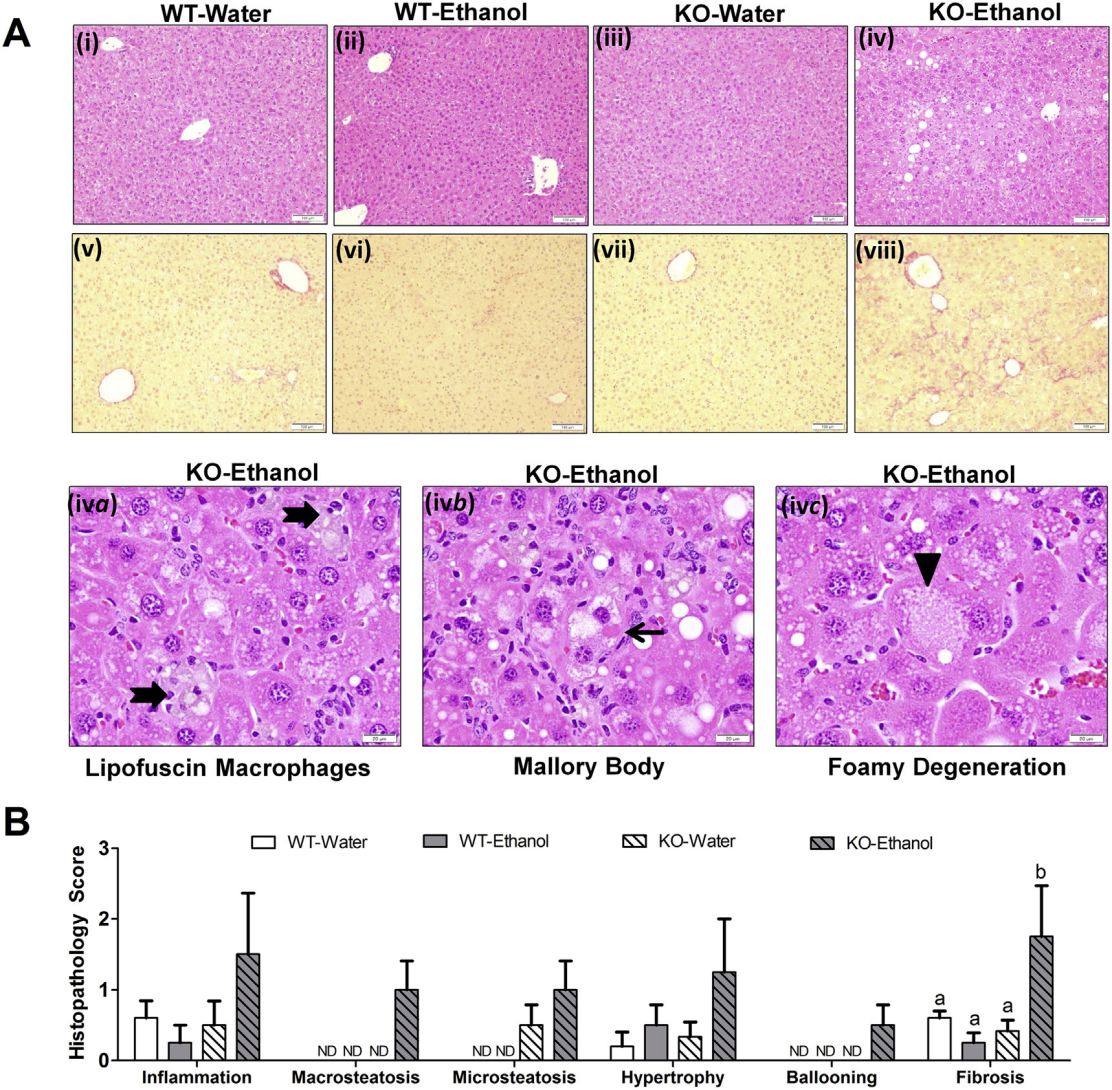
**Ethanol consumption in drinking water leads to evolving hepatic damage in FGF21-KO mice**. After 16 weeks, there are no detectable signs of inflammation, steatosis, and fibrosis in WT-Water mice (A i, A v, B) or WT-Ethanol mice (30% ethanol) (A ii, A vi, B). FGF21-KO-Water also have normal liver histology (A iii, A vii, B). In contrast, FGF21-KO-Ethanol mice drinking 30% ethanol have hepatic damage including inflammation, steatosis, hypertrophy, and fibrosis as indicated by histological analysis (A iv, A viii, B). This includes the presence of hallmark pathological features of alcoholic liver disease such as lipofuscin macrophages (thick arrow) (A iv *a*), Mallory bodies (thin arrow) (A iv *b*), and foamy degeneration of hepatocytes (arrow head) (A iv *c*), only seen in FGF21-KO mice consuming ethanol. *Data represented as Mean* ± *SEM; n* = *5*–*6 mice*/*group. Significance in* (*B*) *was determined with a two-way ANOVA and Bonferroni's post-hoc analysis test for individual comparisons. Significance is designated by letters where means that do not share a common letter are significantly different from each other at P* *<* *0.01. Scale: 100 μm* (*A i–ix*), *20 μm* (*A iv a–c*).

**Supplementary Figure 4:**
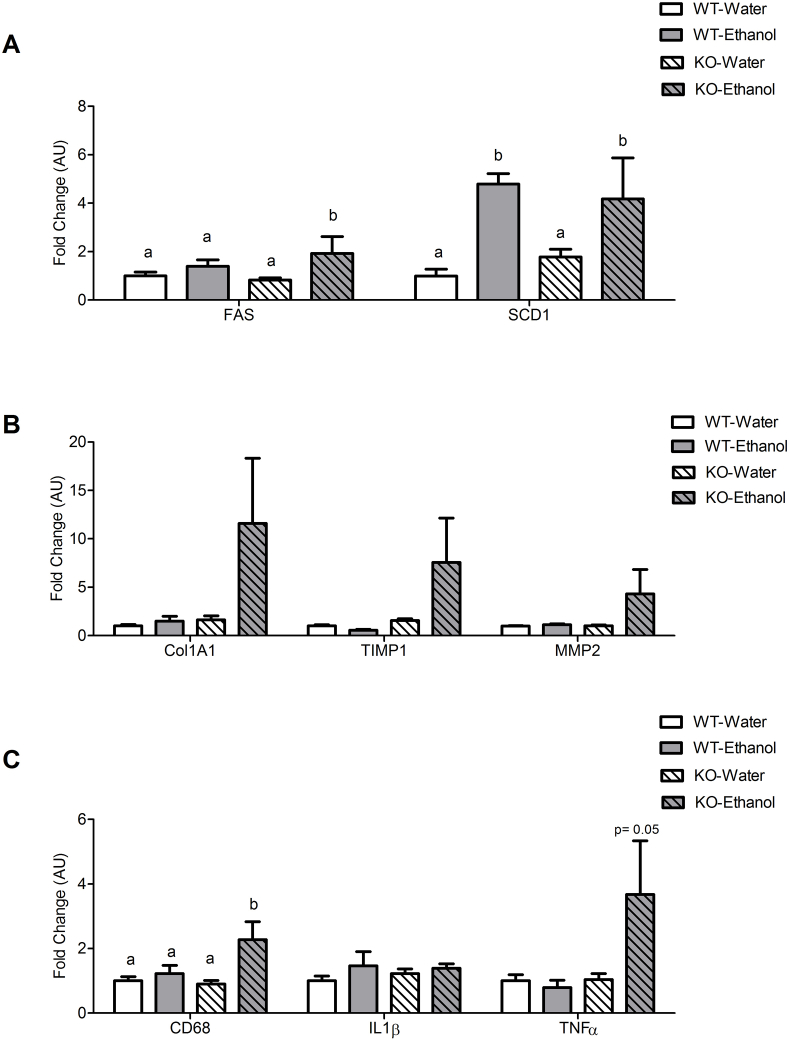
**Lipogenesis, inflammation, and fibrosis markers in mice chronically consuming ethanol in drinking water**. After 16 weeks, WT and FGF21-KO mice consuming 30% ethanol in drinking water along with a chow diet have increased expression of fatty acid synthesis genes FAS and SCD1 (A). In addition, FGF21-KO mice consuming 30% ethanol demonstrate a trend towards increased hepatic expression of fibrotic markers (B) and significant induction of inflammatory markers CD68 and TNFα (C). *Data represented as Mean* ± *SEM; n* *=* *5*–*6 mice*/*group. Significance was determined with a two-way ANOVA and Bonferroni's post-hoc analysis test for individual comparisons. Significance is designated by letters where means that do not share a common letter are significantly different from each other at P* *<* *0.01.*

Although mice appeared grossly healthy at 16 weeks, pathology was observed in the liver. Sections, stained with hematoxylin–eosin were evaluated for histological features of steatosis, inflammation, and hepatocellular injury. Increased inflammation and progressive macro- and micro vesicular steatosis, hypertrophic changes, and ballooning degeneration were noted specifically in the FGF21-KO-Ethanol group compared to other groups (Figure 6A and B i–iv, B). More pronounced fibrosis was observed in FGF21-KO-Ethanol mice compared to other groups as shown by Sirius Red staining (Figure 6A and B v–viii). Hallmark histopathological features of ALD such as lipofuscin laden macrophages (Figure 6A iv *a*), Mallory Bodies (Figure 6A iv *b*) and foamy degeneration (Figure 6A iv *c*) were specifically observed in 50% of FGF21-KO mice. Total triglyceride content in the liver, as measured by Folch Extraction, showed increased accumulation of triglycerides only in the FGF21-KO-Ethanol mice; this was consistent with histological observations (Supplementary Figure 3H). At the level of hepatic gene expression, lipogenic gene FAS was increased in only in the FGF21-KO-Ethanol mice while SCD1 was similarly induced in both WT-Ethanol and FGF21-KO-Ethanol mice compared to mice drinking water (Supplementary Figure 4A).

It is noteworthy that no pathognomonic pathology of ethanol damage was observed on histological analysis of livers from WT-Ethanol mice. Histological markers of ethanol damage were only observed in FGF21-KO-Ethanol mice as noted above. This was consistent with gene expression data as FGF21-KO mice tended to have increased hepatic expression of fibrotic markers such as collagen, type I, alpha 1 (Col1a1), tissue inhibitor of metalloproteinases 1 (TIMP1) and matrix metallopeptidase 2 (MMP2) (Supplementary Figure 4B). They also showed a similar increased trend to hepatic expression of inflammatory markers such as CD68, IL1β, and TNFα (Supplementary Figure 4C).

### 3.5 FGF21 reduces preference for ethanol

**Figure 7:**
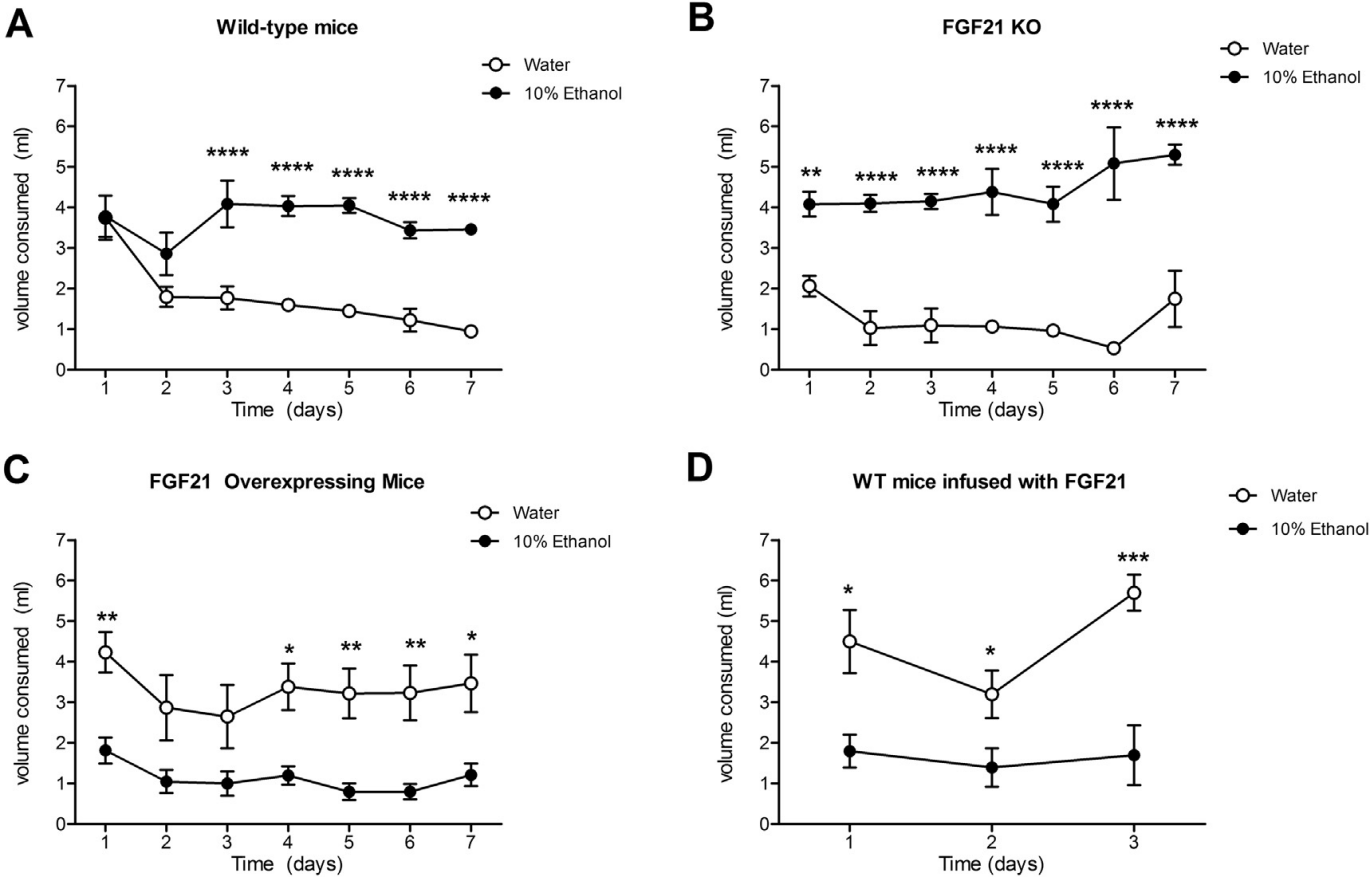
**Excess FGF21 reduces preference for ethanol**. WT and FGF21-KO mice demonstrate a similar strong, immediate preference for 10% ethanol (v/v) over water in a two-bottle preference test (A, B) (WT: *P* < 0.0001; FGF21-KO – *P* < 0.0001). FGF21-OE mice prefer water over ethanol (C). Similarly, WT mice when infused peripherally with FGF21 switch their preference to water from ethanol (D). *Data represented as Mean* ± *SEM; n* = *6 mice*/*group. Significance determined with a two-way ANOVA with repeated measures and Bonferroni's post-hoc analysis test for individual comparisons. Significance is designated by asterisks with* *P < 0.05, **P < *0.01*, ***P < *0.001*, ****P < *0.0001.*

In a two bottle preference test, both WT mice and FGF21-KO mice demonstrated the same, immediate, strong preference for 10% ethanol over water (WT: P < 0.0001; FGF21-KO: P < 0.0001) (Figure 7A and B). By contrast, mice with increased circulating FGF21 levels preferred water over 10% ethanol. This included mice which genetically over-expressed FGF21 (P < 0.01) (Figure 7C) as well as wild type mice infused with FGF21 via subcutaneous osmotic mini-pumps for 3 days (P < 0.05) (Figure 7D).

## 4 Discussion

FGF21 is a complex metabolic regulator that plays a role in both the fed and fasted state [2]. Dissecting the various roles of FGF21 is complicated as it is synthesized by multiple tissues and acts on several target tissues. For example, FGF21 expression is increased in the liver with fasting, fructose challenge, and protein restriction [3], [9], [10]. FGF21 can be induced in inguinal fat depots upon cold exposure [22] and in muscle that is under stress induced by a mitochondrial myopathy [26]. Understanding FGF21 is further complicated by the fact that not all stimuli that induce FGF21 in mice have similar effects in humans. Thus far, fructose challenge [8], [9], protein restriction [10], [27], muscle myopathies [26], fatty liver [4], [6], [7] and pancreatitis [28], [29], [30] are consistent in elevating FGF21 levels in both species.

FGF21 has been shown to have anti-inflammatory and anti-fibrotic effects in the liver and pancreas. FGF21 deficient mice consuming a lipotoxic diet show exacerbated liver damage [31], [32] which can be prevented by exogenous FGF21 infusion. Similarly, FGF21 deficient mice have exacerbated experimental pancreatitis [33], while FGF21-KO mice consuming a conventional obesogenic high fat, high sucrose diet develop exaggerated peri-portal pancreatic inflammation [29].

Ethanol consumption has been reported to induce hepatic transaminases (ALT and AST), alkaline phosphatase, inflammatory markers, fibrotic markers, and carbohydrate induced genes in the liver [34], [35], [36]. While it is known that FGF21 may play a role in ethanol preference in both humans and mice, an acute endocrine response to ethanol consumption in the liver has not been previously reported in either species. Here we show for the first time that acute ethanol consumption induces significant increases in circulating FGF21 in both humans and mice.

Binge ethanol consumption results in a 40-fold induction of circulating FGF21 in humans, substantially higher than the previously reported 4-fold FGF21 induction observed with a fructose challenge in both lean and obese individuals [8] (Supplementary Figure 2A). While an acute fructose challenge in human increases circulating FGF21 within 2 h, ethanol peak values are noted later, typically between 6 h and 9 h after a “binge” episode. Interestingly, the FGF21 time course in response to acute ethanol is similar in mice and humans. Despite the rise in circulating FGF21, we found that FGF21 does not affect ethanol clearance, as mice lacking FGF21 have similar serum ethanol concentrations as well as rates of clearance after ethanol gavage as compared to WT mice.

To investigate the potential role of FGF21 in ethanol induced liver pathology, we evaluated the consequences of chronic ethanol exposure in mice lacking FGF21. In contrast to a previous report [37], we found that chronic ethanol consumption at 6% (w/v) concentration mixed in a high fat LDC diet, was associated with more severe hepatic damage and unexplained early mortality within a period of 16 days in FGF21-KO mice. FGF21-KO-LDC mice had higher levels of serum ethanol compared to WT-LDC mice and this was associated with a reduced expression of ethanol metabolizing genes. Accumulation of ethanol reflects the impaired capacity of FG21-KO mice to oxidize the ethanol in this chronic consumption model. This may be an indirect effect of impaired hepatic function, suggesting a disruption of carbohydrate and lipid metabolizing functions of FGF21.

Given the high rate of morbidity and mortality we observed with this diet, we examined the effect of FGF21 deletion in an alternate model. Chronic ethanol consumption, consumed at 30% (v/v) in drinking water and accompanied by a standard chow diet, did not lead to mortality in FGF21-KO mice over a 16 week period. Nevertheless, hepatic damage was observed in FGF21-KO as reflected by more severe fibrosis and the development of hallmark features of ALD including Mallory bodies, foamy degeneration, and lipofuscin laden macrophages on liver histology [38]. These histological parameters were not observed in WT mice. These changes were accompanied by increased hepatic gene expression of both inflammatory and fibrotic markers in FGF21-KO mice compared to WT mice. It is noteworthy that after 16 weeks of ethanol consumption, WT mice showed no pathologic changes. Thus, ethanol consumption in the absence of high dietary fat led to a gradual development of hepatic damage in FGF21-KO mice. However, in this model, despite evidence of evolving liver damage in FGF21-KO animals, serum ethanol levels were the same as those noted in WT mice. This indicates that at the time point evaluated, both groups of animals had the same capacity to metabolize ethanol and implies that hepatic dysfunction was not yet sufficiently severe to impair ethanol metabolism in the FGF21-KO animals.

We attribute the difference in morbidity and mortality in mice lacking FGF21 between the two diets to the fact that ethanol and high fat diet have a synergistic negative effect on fatty liver disease, an interaction that has been demonstrated in humans [39]. Although the disease progression is different in the two dietary models, both demonstrate hepatic damage in FGF21-KO mice, suggesting a protective role of FGF21 in ethanol induced liver damage.

It might be expected that mice lacking FGF21 would have an aversion to ethanol as consumption ultimately leads to liver damage. In contrast to previously published reports, we observed no difference between WT and FGF21-KO mice in ethanol preference [15]. However, mice overexpressing FGF21 had reduced ethanol consumption compared to WT mice, consistent with previous reports [40]. This effect was also observed when WT were infused subcutaneously with FGF21, which led to a reversal of preference for ethanol over water during the course of the infusion.

Our data suggest that FGF21 has a dual role in ethanol metabolism. Acutely, FGF21 acts centrally to inhibit ethanol consumption and the acute rise in FGF21 in both rodents and humans may mediate this function. Chronically, the rise in hepatic FGF21 expression may serve the now well-established hepatic anti-inflammatory and anti-fibrotic role, in part by leading to increased fatty acid oxidation and limiting lipid accumulation [31], [32]. Future studies are required to explore these actions of FGF21 further. Taken together, these effects suggest the potential value of FGF21 as a therapeutic agent for alcoholic liver disease [41].

## Appendix A Supplementary data

Supplementary Table 1 Inclusion and exclusion criteria for the enrollment of human subjects and restrictions applied before study. Supplementary Table 1

Supplementary Table 2 Demographics of human subjects. Supplementary Table 2
