# Supplementary material for: Fibroblast growth factor 21 (FGF21) is robustly induced by ethanol and has a protective role in ethanol associated liver injury
Source: Mol Metab. 2017 Aug 19;6(11):1395–406. doi: 10.1016/j.molmet.2017.08.004 (PMC5681240; doi:10.1016/j.molmet.2017.08.004)
Supplement: Supplementary Table 1 — Inclusion and exclusion criteria for the enrollment of human subjects and restrictions applied before study. [file mmc1.pdf]

Supplementary TABLE 1

Inclusion/Exclusion Criteria - Humans

Inclusion Criteria	
Age	21 – 60 years
BMI	< 25
Smoking	No
Contraception	Yes, both males and females
Vitamins	Stop prior to enrollment
Ethanol	No

Exclusion Criteria
Nursing
Experimental Drugs:<br>No drugs 4 weeks prior, no blood donation 8 weeks prior to enrollment
Coagulation/Bleeding Disorders
Nutritional inefficiencies (specifically for Fe, Zn, Cu, Mg)
Fish Allergies
Cancer/Cardiovascular/Renal/Hepatic/Respiratory/Endocrine/Neurologic disease
High Blood Pressure<br>Systolic BP > 160 - No<br>Diastolic BP > 95 – No
GI Disorders
History of ethanol consumption:<br>More than 3 drinks/day

Restrictions before Study
Subjects were advised to abstain from the following:<br>i) caffeine or high fat foods 24 hours prior to each study visit<br>ii) high doses of antioxidant vitamins daily (vitamin C > 1000mg, Vitamin E > 400 IU, Beta Carotene > 1000IU, Vitamin A > 5000IU, Selenium > 200mcg, Folic Acid > 1mg) for 2 weeks prior to the start of the study and throughout the study<br>iii) acetaminophen, NSAIDS, COX-2 inhibitors (OTC or prescription) for 1 week prior to the start of the study and throughout the study<br>iv) alcohol consumption 24 hours prior to the start of each study visit and throughout the active study (unless part of the study intervention)<br>v) medications including antibiotics 2 weeks prior to the start of the study and throughout the study (birth control pills were acceptable)
