# Supplementary material for: Fibroblast growth factor 21 (FGF21) is robustly induced by ethanol and has a protective role in ethanol associated liver injury
Source: Mol Metab. 2017 Aug 19;6(11):1395–406. doi: 10.1016/j.molmet.2017.08.004 (PMC5681240; doi:10.1016/j.molmet.2017.08.004)
Supplement: Supplementary Table 2 — Demographics of human subjects. [file mmc2.pdf]

Supplementary TABLE 2

Humans - Demographics

Gender	Age (yr)	Weight (kg)	Height (cm)	Ethnicity	Race	Treatment
Male	31	55.9	168.7	Non Hispanic	Caucasian	Supplement Study
Female	53	54.6	152.4	Hispanic	Caucasian	Supplement Study
Female	28	54.6	160.0	Non Hispanic	Caucasian	Supplement Study
Male	46	75.2	173.5	Non Hispanic	Caucasian	Supplement Study
Female	54	73.1	172.7	Non Hispanic	Caucasian	Supplement Study
Male	50	118.5	196.1	Hispanic	Caucasian	Supplement Study
Female	27	68.5	170.2	Non Hispanic	Caucasian	Supplement Study
Female	56	53.3	152	Hispanic	Caucasian	EtOH 0.9g/Kg
Male	49	77.7	173	Non Hispanic	Caucasian	EtOH 0.9g/Kg
Female	58	64	162	Non Hispanic	African-American	EtOH 0.9g/Kg
Male	30	80	185	Non Hispanic	African-American	EtOH 0.9g/Kg
